# Spread of yellow fever virus outbreak in Angola and the Democratic Republic of the Congo 2015–16: a modelling study

**DOI:** 10.1016/S1473-3099(16)30513-8

**Published:** 2017-03

**Authors:** Moritz U G Kraemer, Nuno R Faria, Robert C Reiner, Nick Golding, Birgit Nikolay, Stephanie Stasse, Michael A Johansson, Henrik Salje, Ousmane Faye, G R William Wint, Matthias Niedrig, Freya M Shearer, Sarah C Hill, Robin N Thompson, Donal Bisanzio, Nuno Taveira, Heinrich H Nax, Bary S R Pradelski, Elaine O Nsoesie, Nicholas R Murphy, Isaac I Bogoch, Kamran Khan, John S Brownstein, Andrew J Tatem, Tulio de Oliveira, David L Smith, Amadou A Sall, Oliver G Pybus, Simon I Hay, Simon Cauchemez

**Affiliations:** a Department of Zoology, University of Oxford, Oxford, UK; b Institute for Health Metrics and Evaluation, University of Washington, Seattle, WA, USA; c Oxford Big Data Institute, Li Ka Shing Centre for Health Information and Discovery, Oxford, UK; d School of BioSciences, University of Melbourne, Parkville, VIC, Australia; e Mathematical Modelling of Infectious Diseases and Center of Bioinformatics, Biostatistics and Integrative Biology, Institut Pasteur, Paris, France; f Centre National de la Recherche Scientifique, URA 3012, Paris, France; g Health Programme, European Commission, International Cooperation and Development, Delegation en RDC, Kinshasa, Democratic Republic of the Congo; h Centers for Disease Control and Prevention, San Juan, PR, USA; i Center for Communicable Disease Dynamics, Harvard T H Chan School of Public Health, MA, USA; j Harvard University Medical School Boston, MA, USA; k Department of Epidemiology, Johns Hopkins Bloomberg School of Public Health, Baltimore, MD, USA; l Arbovirus and Viral Hemorrhagic Fever Unit, Institut Pasteur da Dakar, Dakar, Senegal; m Environmental Research Group Oxford, Department of Zoology, Oxford, UK; n Robert Koch Institut, Berlin, Germany; o Research Institute for Medicines (iMed.ULisboa), Faculty of Pharmacy, University of Lisbon, Portugal; p Centro de Investigacao Interdisciplinar Egas Moniz, Instituto Superior de Ciencias da Saude Egas Moniz, Caparica, Portugal; q Computational Social Science, ETH Zurich, Zurich, Switzerland; r School of Medicine, University of California San Francisco, San Francisco, CA, USA; s Divisions of General Internal Medicine and Infectious Diseases, Toronto General Hospital, University Health Network, Toronto, ON, Canada; t Li Ka Shing Knowledge Institute, St Michael's Hospital, Toronto, ON, Canada; u WorldPop, Department of Geography and Environment, University of Southampton, Southampton, UK; v Flowminder Foundation, Stockholm, Sweden; w School of Laboratory Medicine and Medical Sciences, Nelson R Mandela School of Medicine, College of Health Sciences, University of KwaZulu-Natal, Durban, South Africa; x Sanaria Institute for Global Health and Tropical Medicine, Rockville, MD, USA

## Abstract

**Background:**

Since late 2015, an epidemic of yellow fever has caused more than 7334 suspected cases in Angola and the Democratic Republic of the Congo, including 393 deaths. We sought to understand the spatial spread of this outbreak to optimise the use of the limited available vaccine stock.

**Methods:**

We jointly analysed datasets describing the epidemic of yellow fever, vector suitability, human demography, and mobility in central Africa to understand and predict the spread of yellow fever virus. We used a standard logistic model to infer the district-specific yellow fever virus infection risk during the course of the epidemic in the region.

**Findings:**

The early spread of yellow fever virus was characterised by fast exponential growth (doubling time of 5–7 days) and fast spatial expansion (49 districts reported cases after only 3 months) from Luanda, the capital of Angola. Early invasion was positively correlated with high population density (Pearson's *r* 0·52, 95% CI 0·34–0·66). The further away locations were from Luanda, the later the date of invasion (Pearson's *r* 0·60, 95% CI 0·52–0·66). In a Cox model, we noted that districts with higher population densities also had higher risks of sustained transmission (the hazard ratio for cases ceasing was 0·74, 95% CI 0·13–0·92 per log-unit increase in the population size of a district). A model that captured human mobility and vector suitability successfully discriminated districts with high risk of invasion from others with a lower risk (area under the curve 0·94, 95% CI 0·92–0·97). If at the start of the epidemic, sufficient vaccines had been available to target 50 out of 313 districts in the area, our model would have correctly identified 27 (84%) of the 32 districts that were eventually affected.

**Interpretation:**

Our findings show the contributions of ecological and demographic factors to the ongoing spread of the yellow fever outbreak and provide estimates of the areas that could be prioritised for vaccination, although other constraints such as vaccine supply and delivery need to be accounted for before such insights can be translated into policy.

**Funding:**

Wellcome Trust.

## Introduction

Yellow fever virus is a mosquito-borne flavivirus that causes infections in human beings, with symptoms ranging from mild non-specific illness to severe disease with jaundice, haemorrhage, and death.^1^ A single-dose vaccine has existed since the 1940s and has helped to control and reduce yellow fever virus transmission substantially.^2^, ^3^, ^4^ Complete eradication is, however, prevented by the sylvatic cycle of the virus within which non-human primates act as primary hosts and *Aedes aegypti* mosquitoes are responsible for occasional transmission to people.^5^, ^6^

From Dec 5, 2015, to November, 2016, a large yellow fever outbreak has affected Angola and the Democratic Republic of the Congo (DR Congo), with 7334 suspected cases, of which 962 have been confirmed, and 393 deaths reported to WHO as of Oct 28, 2016.^7^ Responses to such outbreaks rely mainly on reactive vaccination campaigns and pose various strategic and logistical challenges. For example, in response to the current outbreak in central Africa, the global yellow fever virus vaccine emergency stockpile (6 million doses) was exhausted after the initial mass vaccination campaign.^7^ In the context of finite resources, decisions about which geographic areas should be targeted first need to be informed by a detailed understanding of the determinants of the spatial spread of yellow fever virus and by predictions of where yellow fever virus is most likely to spread to in the future.

Such assessments need to capture a range of factors. First, human mobility could facilitate the introduction of the pathogen into disease-free areas, which has been reported for other outbreaks in Africa.^8^, ^9^ Such regional spread of a disease is largely governed by underlying population structures and transport networks, as well as by individuals' economic, cultural, and recreational activities.^10^, ^11^ Furthermore, in the context of a vector-borne disease such as yellow fever, the ecological landscape of the *A aegypti* mosquito that transmits the virus between people must also coincide with patterns of human movements for a successful viral transmission cycle to be established.^12^, ^13^ This ecological landscape has been shown to be strongly affected by temperature, precipitation, humidity, vegetation coverage, and degree of urbanisation.^14^

**Research in context**

**Evidence before this study**

We searched PubMed up to Oct 11, 2016, for publications about any yellow fever virus outbreaks in Angola and the Democratic Republic of the Congo (DR Congo), yellow fever virus outbreaks since the beginning of 2015, and the spatial spread of yellow fever virus, without any language restrictions. We used the search terms (“yellow fever”[MeSH Terms] OR “yellow fever”[All Fields]) AND ((“angola”[MeSH Terms] OR “angola”[All Fields]) OR (“congo”[MeSH Terms] OR “congo”[All Fields] OR “DRC”[All Fields]) OR ((“epidemic”[All Fields] OR “epidemics”[MeSH Terms] OR “disease outbreaks”[MeSH Terms]) AND (“2015/01/01”[PDAT]:”3000/12/31”[PDAT])) OR “spatial”[All Fields]). Almost no empirical information is available for the current yellow fever virus outbreak in Angola and DR Congo. Previous work has described the identification of the outbreak, discussed vaccination coverage and the apparent vaccine shortage from a policy perspective, and emphasised the risk of international spread outside the region.

**Added value of this study**

To our knowledge our study is the first to investigate key epidemiological parameters of the outbreak and show the important ecological and demographic determinants that govern transmission and spread of the virus in the region.

**Implications of all the available evidence**

Across districts in Angola and the DR Congo, the spread of yellow fever virus was governed by high population density, including locations that were distant from the origin of the outbreak in Luanda. Transmission is also more likely to be sustained in areas where people live in close proximity. Human movements in and out of the capital cities of Angola and the DR Congo have caused yellow fever virus to spread to almost all districts in Angola. Our model fits the expansion process of the pathogen well and allows for extrapolation into the future. Our approach can be used to help policy makers prioritise areas to be targeted, especially in the context of finite public-health resources.

In this study, we jointly analysed datasets describing the epidemic and spatial spread of yellow fever virus, vector suitability, human demography, and mobility in central Africa, aiming to better understand, quantify, and predict the spread of yellow fever virus in this region. By identifying the areas that are at the highest risk of yellow fever virus transmission, such a framework could inform the response to ongoing outbreaks.

## Methods

### Epidemiological and vector data

We obtained epidemiological data on confirmed and suspected yellow fever virus cases in Angola and the DR Congo (the most affected countries) from the WHO situation reports for the period Dec 4, 2015, to Aug 4, 2016. We extracted the first and last dates of disease onset for each district in Angola (n=163) and for each commune in the DR Congo (n=150). We used numbers of cases per week in Angola, but weekly case numbers were not available for the DR Congo at the time of data collection (Aug 4, 2016). We matched locations with administrative unit files available from the Database of Global Administrative Areas.

To generate district-level ecological risk of disease transmission, we extracted estimated suitability values for the primary urban vector *A aegypti* at the lowest administrative level (ie, all 313 districts in Angola and communes in the DR Congo).^14^, ^15^ These estimates combine climatic and socioeconomic variables to produce an estimate of vector suitability and have previously been used to estimate the time-varying risk of the emergence and spread of arboviruses.^16^, ^17^

### Human movement data and models

We considered eight connectivity metrics in our model, all of which capture different aspects of connectivity between two districts, shown to be useful for inferring regular daily commuting patterns, longer-term movements, and the general human diffusion process.^8^, ^18^ The first set of metrics included distance and travel time distance. For distance, we calculated the great circle distance between the centroids of each pair of districts using data from using data from the Database of Global Administrative Areas. For travel time distance, we calculated the travel time between each pair of districts.^19^ This metric represents the underlying transport network, which has been shown to reflect population mixing (appendix p 7).^16^

We derived a second set of metrics from standard models describing human mobility. These were the gravity model, the radiation model, and adjacency network metrics. The gravity model assumes that the relative population flow between districts is a log-linear function of the population sizes of the districts and the distance between them (functional form shown in the appendix).^20^ This model therefore emphasises the attractive power of large population centres such as the capital cities Luanda and Kinshasa (appendix p 6). The radiation model additionally takes into account the draw from other populations within the same radius of the districts considered, as well as the population sizes and distance of the origin and destination locations (functional form shown in the appendix).^18^, ^21^ This model therefore reflects recurrent workplace commuting, assuming that every locality has a competing underlying attractiveness. Adjacency network metrics encode the number of district borders an individual would need to cross to move from one district to another. This metric thus reflects the effects of national and sub-national borders on movement within the region. We then disaggregated this adjacency matrix into three binary connectivity matrices with connectivity degrees of one (ie, districts that share a border), connectivity degrees of two (ie, districts that share a common neighbour), and connectivity degrees of more than two. A full list of the 11 variables used to predict the geographic expansion of yellow fever virus is available in the appendix (p 8).

To calibrate the gravity and radiation models, we used aggregated and deidentified mobile phone-derived mobility estimates at the constituency level from Namibia between Oct 1, 2010, and Sept 30, 2011. These data measure the proportion of time that unique subscriber identity module (SIM) cards in each constituency spend in all other constituencies, and have been described in detail by Ruktanonchai and colleagues.^22^ We used data from Namibia because it directly neighbours the study area and has a similar per capita gross domestic product (GDP) to both Angola and the DR Congo and because mobile phone-derived mobility estimates were not available from the study region at the time of analysis. We then used the models to predict between-district mobility for Angola and the DR Congo using the movement package in R. We computed national adjacency networks using administrative boundary data from the Database of Global Administrative Areas dataset. For each of the 313 administrative regions in Angola and the DR Congo, we calculated the total human population size using gridded population estimates based on data from WorldPop.

We tested the hypothesis that the rate of spatial expansion changed with time by introducing a time-varying binary variable that was equal to 0 before change point *T* and equal to 1 after *T*. In our baseline scenario, we assumed that the week of the change point corresponded to the rollout of mass vaccination in the region (week 9 of 2016), but we also explored alternative scenarios in a sensitivity analysis.

### Model of geographical spread of yellow fever virus

In our model, *x*_i_*(t)* represents the infection status of district at time (ie, a binary variable that takes the value 1 if there were infections that time step and is 0 otherwise). We assume that districts were infected in all time-steps between the first and last reported cases described in the WHO reports. We used a standard logistic model to characterize the probability that district *j* will become infected at time *t*:

$$logit(P(x_{j}(t)=1|x_{j}(t-1)=0))=\beta_{0}+\sum_{k=1}^{n}\beta_{k}Y_{j,t}^{k}+ɛ$$

*Y*^k^ corresponds to explanatory variable *k* and ε is an error distributed by the standard logistic distribution. Explanatory variables included in this analysis are vector suitability and whether infection occurred after vaccine rollout. Connectivity metrics that quantifies the connectivity between districts *i* and *j* were denoted as follows:

$$A_{ij}^{\left(k\right)}$$

For each of these metrics, we derived the global force of infection exerted from all infected districts to *j*:

$$Y_{j,t}^{k}=\sum_{i}A_{ij}^{\left(k\right)}x_{i}(t-1)$$

We used our model for the geographic spread of yellow fever virus to investigate the contribution of single variables in a univariate analysis and then in a multivariable framework.

To assess the model accuracy, we calculated the probability of invasion *p*_W_ predicted by the model for each district-week that had not yet reported cases. We then partitioned district–weeks into eight groups with predicted probability in the range 0–1%, 1–5%, 5–10%, 10–15%, 15–20%, 20–25%, 25–35%, and 35–100%. For each group, we calculated the mean predicted probability and compared it with the proportion of district–weeks in the group in which invasion effectively took place. Because there are 4·33 weeks per month, the monthly probability of invasion can be calculated with the following formula:

$$p_{M}=1-{\left(1-p_{w}\right)}^{4.33}$$

### Application of the model

In the context of finite resources, we assessed how this analytical framework could have helped to inform the prioritisation of districts to be targeted for intervention at the time when such insight was needed—ie, on week 9 of 2016 when the vaccination campaign started. We fitted the model to data available up until that week, then ran 1000 simulations of the model to infer the probability of invasion in the subsequent month for each district. In each simulation, a district can become infected in a certain week and then contribute to invasion risk in that particular simulation. We assumed that invaded districts would not become disease-free within the short simulation period. Assuming that only *n* districts can be targeted for intervention, we compared the performance of a prioritisation strategy that targeted the *n* districts with the highest predicted invasion probability versus a strategy that targeted *n* districts chosen at random.

For forward prediction, we used the same approach to predict the possible expansion of yellow fever virus 1–2 months ahead. We fitted the model to the entire epidemic from week 49 in 2015 to late August, 2016, then used the model parameters to simulate the geographic spread forward in time.

We estimated the exponential growth rate and the doubling time of the epidemic in the early phase when the number of cases was growing exponentially. We reconstructed the generation time distribution of yellow fever virus (ie, the time lag from the infection of a case to infection of subsequent cases in the chain of transmission) from previous knowledge of the natural history of yellow fever virus infection in people and in mosquitoes. We then derived the reproduction number from standard formula linking it to the exponential growth rate and the generation time distribution.^23^ Technical details are available in the appendix.

Finally, we assessed the sensitivity of our model to time-varying reporting rates. Our spatial model is based on a detailed analysis of the dates when districts reported their first yellow fever virus case. If reporting of cases increased during the course of the epidemic, the delay between the first infection and the first reported case should have shortened with time. We therefore did a simulation study in which, for each district, the date of the first infection was reconstructed from the date of the first report under the assumption of an average delay of 4 weeks in the early phase of the epidemic (until week 9 of 2016) and of 1 week in subsequent weeks.

### Role of the funding source

The funder of the study had no role in study design, data collection, data analysis, data interpretation, or writing of the report. The corresponding author had full access to all the data in the study and had final responsibility for the decision to submit for publication.

## Results

**Figure 1:**
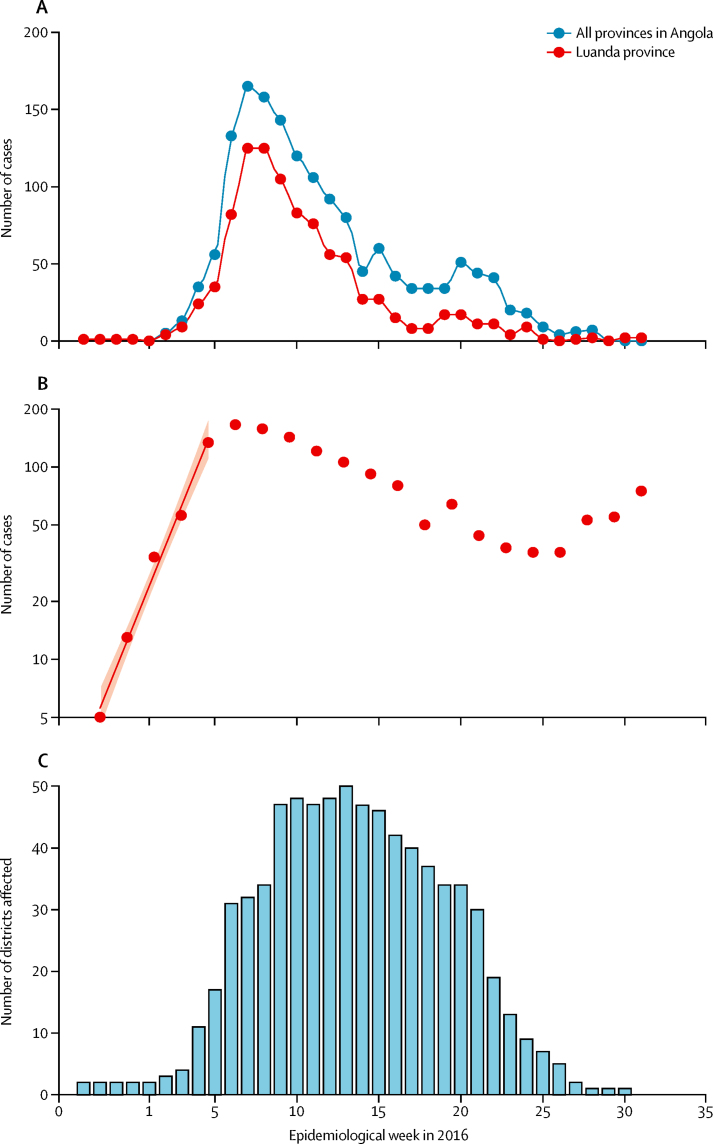
Number of cases in Angola and geographic spread of the epidemic (A) Epidemic curve for suspected and confirmed cases in Angola and Luanda from Dec 1, 2015, to Aug 25, 2016. (B) Fit of exponential growth during the early phase of the epidemic. (C) Number of districts affected during each week over the course of the outbreak.

The first cases of yellow fever were reported in Luanda, the capital of Angola, in the first week of December, 2015. Between week 1 and week 5 of 2016, the number of cases grew exponentially, with a doubling time of 6 days (95% CI 5–7; figure 1A). Under the assumptions that reporting remained stable during this period and that the generation time had a mean of 15 days (SD 6; appendix), we estimated that the reproduction number of yellow fever virus was 4·8 (95% CI 4·0–5·6).

**Figure 2:**
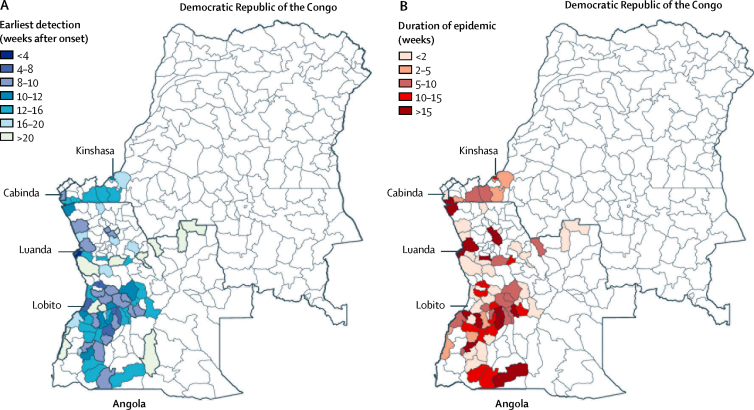
Timing of the introduction of yellow fever virus and duration of infection (A) Timing of the introduction of yellow fever virus to each district starting from the origin of the outbreak in Luanda, Angola. Colouring shows the weeks until the first case was reported. (B) Duration of transmission.

Although the epidemic was initially focused in Luanda, it quickly expanded to other districts, including Belas, Lobito, and Huambo (figure 1B and figure 2A). By the end of February, 49 districts had reported cases (figure 1B) with the proportion of cases from Luanda dropping from 75% in early February, 2016 (week 5), to about 30% in April, 2016 (week 14; figure 1A).

In a simple univariate correlation analysis, we found that earlier timing of yellow fever virus invasion into a district was correlated with greater population density (Pearson's *r* 0·52, 95% CI 0·34–0·66; appendix pp 6, 9). Additionally, the further away districts were from Luanda by distance or travel time, the longer they took to be invaded (Pearson's *r* for distance 0·60, 0·52–0·66; Pearson's *r* for travel time 0·63, 0·56–0·69; appendix p 10). By contrast, early invasion was not correlated with *A aegypti* suitability, which is abundant in most of the study area (appendix p 13).^14^

We also investigated the duration that the virus persisted in a district after invasion. In a survival analysis, the probability that a district was still reporting cases was 0·94 (95% CI 0·84–1·00) at 17 weeks after the first report, 0·88 (0·73–1·00) at 18 weeks, and 0·50 (0·26–0·98) at 20 weeks (appendix p 9). In a Cox model, we estimated that the hazard ratio for cases ceasing was 0·74, 95% CI 0·13–0·92, per log-unit increase in the population size of a district. The duration for which a district reported cases was not significantly associated with the date when it was first invaded.

**Figure 3:**
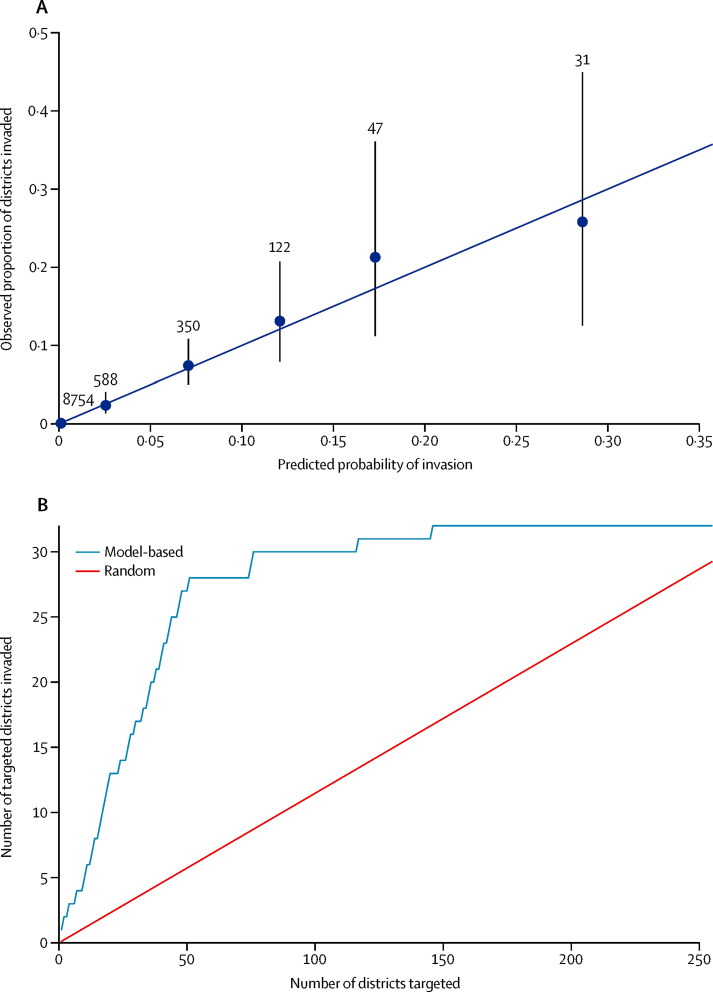
Model accuracy and real-time prediction of the yellow fever virus invasion model (A) Model prediction accuracy as assessed by comparing the predicted invasion probability from the geographic spread model with the observed proportion of districts that became invaded; numbers represent district–weeks. (B) Comparisons between district targeting based on real-time modelling analysis *vs* random targeting during the expansion phase of the outbreak between mid-March and mid-April, during which 32 districts were newly invaded.

We then fitted the geographic spread model to all data available (ie, Dec 4, 2015, to Aug 4, 2016). The terms retained in the model were, first, the adjacency metric (more than two degrees away); second, the binary variable defining whether the invasion occurred before or after vaccination rollout at week 9 of 2016; third, *A aegypti* suitability; fourth, the gravity human mobility metric; and fifth, the radiation human mobility metric. In our assessment of model accuracy, we found excellent agreement between the probability of invasion per week per district, as predicted by the model, and the observed proportion invaded (figure 3A). The model successfully discriminated district–weeks with a high probability of invasion from district–weeks with a lower probability of invasion (area under the curve 0·94, 95% CI 0·92–0·97; appendix p 16). For example, 31 (0·31%) district-weeks had a predicted weekly probability of invasion greater than 25%, corresponding to an average per month probability of invasion of 77% (figure 3A). By contrast, 8754 (88·5%) district–weeks had a weekly invasion probability less than 1% (figure 3A; appendix p 11). If we judge the expanding phase of the outbreak to be from late January to late March, then there was also a high correlation between the invasion of yellow fever virus and the invasion probability predicted by the model (0·78, 95% CI 0·74–0·82).

We noted that spatial expansion slowed down significantly during the course of the epidemic (figure 1B). If we assumed that the change occurred on week 9 of 2016, when vaccination was rolled out, the odds ratio for a district being invaded after the change point *T* versus before *T* was 0·948 (95% CI 0·946–0·949; appendix pp 17, 18). However, the proportion of deviance explained was slightly higher if we used the assumption that the change occurred 2–3 weeks before vaccination roll out (33·6% for 2 or 3 weeks before week 9 *vs* 30·8% for week 9; appendix p 18).

In the context of finite resources, insights into district-specific real-time invasion risks might help to inform the prioritisation of districts to be targeted for interventions such as vaccination and vector control. As an example, we considered the situation in mid-February, 2016, when the epidemic was still expanding (32 districts were invaded in the next month). If, on the basis of data available up to mid-February, the 20 districts with the highest probability of invasion had been targeted for vaccination, then 13 (41%) of the 32 districts that were actually invaded would have been targeted, whereas this number would have increased to 17 (53%) if the 30 districts with the highest risk had been targeted and 27 (84%) if the 50 districts with the highest probability had been targeted (figure 3B; appendix p 12). The number of districts successfully targeted would drop to two (6%), three (9%), and six (19%), respectively, if prioritisation had been random.

Univariate models tended to be worse than the full model when we compared the models' predicted probability of invasion versus the observed proportion of districts invaded (figure 3A; appendix p 11). However, we were encouraged to see that simpler models that included only travel distance or neighbourhood effects were able to discriminate similarly well in terms of their ranking of locations that might become affected (appendix p 14).

**Figure 4:**
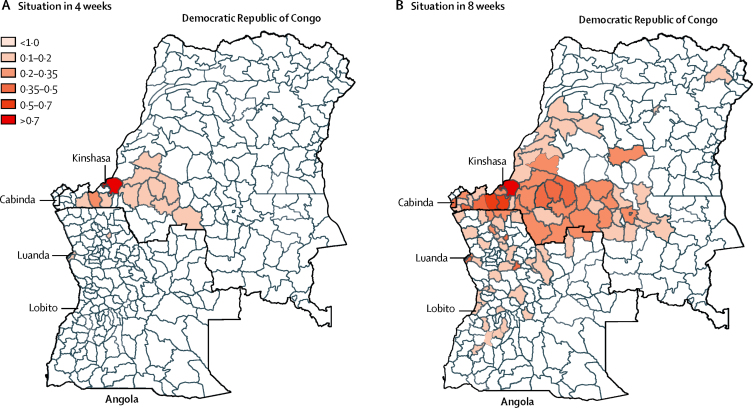
Model-based predictions of yellow fever virus spread Maps show model-based predictions for the invasion of yellow fever virus in central Africa originating from Kinshasa, the location with the latest reported cases, at 4 weeks (A) and 8 weeks (B) ahead of the last case onset date, July 12, 2016. Colours represent weekly probability of invasion.

We then used the full model to predict the future spread of yellow fever virus in Angola and the DR Congo (figure 4). The most recent confirmed cases of yellow fever are from Kinshasa in the DR Congo, and we predicted that future spread would mostly occur along the road east to Kananga and south to the border with Angola where cases have been identified previously (figure 4; appendix pp 6, 7). The two locations in Angola with the highest predicted risk of introduction are Uige and Luanda (figure 4). Rural regions in Angola and the DR Congo had low predicted probabilities of yellow fever virus introduction.

We investigated the robustness of the variables retained in the final model to the time when the analysis was done. The gravity metric was the variable selected most often at 21 out of 24 weeks compared with 17 times for the radiation metric and 19 times for the aedes suitability surface (appendix p 15). However, there was heterogeneity as to when these variables were selected. For example, the gravity metric seemed to be important in the early phase of the outbreak and radiation metric seemed to be important in the transitioning phase. In a sensitivity analysis, we noted that model parameters would remain almost identical and the predictive accuracy would remain very high if the delay from the first infection to the first reported case in a district had shortened from 4 weeks to 1 week during the course of the epidemic (appendix pp 16, 17).

## Discussion

In the context of rapidly spreading and potentially fatal infectious disease epidemics for which vaccine stockpiles are limited, such as the current yellow fever outbreak in central Africa, it is essential to establish which areas are at the greatest risk of infection to inform vaccine prioritisation decisions. This risk estimation requires an in-depth understanding of the determinants of disease spread. We integrated and analysed diverse datasets, including the size and mobility of human populations and detailed maps of vector suitability, to describe the epidemic and spatial spread of yellow fever virus. We found that the spatial spread of yellow fever virus was well explained by human mobility and vector suitability and that our approach could help to discriminate districts at high risk of invasion from others at lower risk.

We estimated that the reproduction number of yellow fever virus was 4·8, which is in line with other estimates available for yellow fever virus.^24^ This suggests a critical vaccination coverage for yellow fever virus of about 80%. However, we cannot rule out the possibility that in the early stage of the epidemic, part of the rise in case counts was caused by increased reporting, which could bias estimates of the reproduction number (and the critical vaccination coverage) upwards. Although we estimated the reproduction number from national data, it will be interesting to assess transmission dynamics at a refined local level.

We characterised various factors that affect the spread of yellow fever virus in central Africa. For example, we found that human mobility was an important predictor of the spatial expansion of yellow fever virus. The importance of large population centres in driving the expansion dynamics during the early stage of the epidemic was apparent in both the univariate and multivariate models, and the gravity metric played a key part early in the epidemic (figure 2A and 2B; appendix p 6). Our results from the univariate analysis also suggest that a substantial neighbourhood effect existed during this outbreak—ie, places closer to the outbreak were more likely to become infected than were those further away, which was exemplified by the adjacency and travel distance metric models performing better than the other univariate models (appendix p 11). Univariate models were not well suited to capture the dynamics of the spread that include short and long distance introductions of the pathogen, such as the cases reported in Kinshasa, DR Congo, most of which are connected to Luanda, Angola. The inclusion of the spatial distribution of *A aegypti* suitability substantially increased the fit of the full model, but this variable did not show significant associations with early yellow fever virus invasion (appendix p 11). Vector suitability is mostly constant across the region, which might result in a lower predictive power for that variable (appendix p 13).^14^ More studies are needed to measure the mosquito per human ratio in Angola and the DR Congo and to quantify their spatial heterogeneity (appendix p 13). Our data also suggested that epidemics might last longer in areas that are more densely populated (figure 2B; appendix p 6). This effect would support estimates of other arboviruses such as dengue virus, for which urban areas have been shown to confer substantially higher reproduction numbers because of a higher person-to-person contact rate.^16^

In our analysis, the rate of spatial expansion of yellow fever virus declined in February, 2016, which roughly coincides with the start of the vaccination campaign. Although the observation of such temporal association is interesting, our statistical framework does not allow us to show a causal link or to quantify the proportion of the decline that was attributable to vaccination versus other causes. There is weak evidence in the data that spatial expansion might have started to decline even before vaccination started (appendix p 18). However, the current absence of data describing the vaccination campaign makes more detailed and a definitive assessment of its effects on spread difficult. Future research should aim to further characterise this impact from more detailed data documenting both case counts and vaccine distribution over space and time. This future work should assess the impact of vaccination on both spatial expansion (ie, ability of yellow fever virus to spread from district to district) and on local transmission (ie, ability of yellow fever virus to generate outbreaks within districts).

In addition to understanding the drivers of the spread of yellow fever virus transmission, we tested our statistical model's predictive power in real time. During the expanding phase of the outbreak, the model could help to predict the invasion of yellow fever virus in the region and to identify which districts should be targeted for intervention (figure 3B). This result is useful in a context in which resources and vaccine stock are finite. We emphasise the need to reparameterise the model because the importance of different ecological and mobility factors might vary depending on the week of the epidemic. Fortunately, the outbreak is now contained and no confirmed cases have been reported since July 12, 2016.^7^ Nevertheless, our approach presents a flexible framework that can be readily updated and could therefore be used to predict the geographic spread of future epidemics of yellow fever virus or other related infectious diseases. However, to translate the insight generated by such a modelling approach into a concrete vaccination strategy, complex logistic constraints would also need to be accounted for, such as supply and delivery. These important issues are beyond the scope of this study. Our estimates of characteristics of spatial spread might be affected if the reporting of cases varies spatially.

During the past 10 years or so, mosquito-borne diseases such as those caused by dengue virus, chikungunya virus, and more recently Zika virus, have been expanding geographically. By contrast, yellow fever virus was previously thought to be on the decline because of the positive effects of vaccination campaigns. However, the recent transmission of yellow fever virus in central Africa has shown the continued threat that yellow fever virus poses to human populations, as well as the need for continued vaccination campaigns and close monitoring of sporadic outbreaks in rural regions. Previous work has also shown that vaccination coverage in the region analysed here is low and that there was no significant spatial heterogeneity between districts.^6^ In the wider context, increased urbanisation and human mobility in the region could lead to future increases in the risk of emergence and rapid expansion of such outbreaks as the current yellow fever epidemic.

At this stage, because of the absence of available data, our analysis cannot address the yellow fever virus outbreak from a genetic perspective. However, our results provide a baseline to which future viral genetic results could be directly added. Genomic surveillance could be used to extend our results, by helping to identify the origin of the outbreak,^25^ monitoring the diversity and spatial distribution of circulating viruses,^26^ and characterising signatures of host adaptation.^27^ Two past outbreaks of yellow fever virus have been recorded in Angola, in 1971 and 1988, and a single genetic isolate indicates that the 1971 outbreak strain belongs to the east and central African genotype. Although genetic data for the current outbreak in central Africa have yet to be reported, preliminary analysis of available yellow fever virus genomes from travellers returning from the affected region suggest that the current outbreak also belongs to the east and central African lineage.^28^ Whether the virus has been circulating in a sylvatic cycle in rural areas, or whether cryptic circulation has been occurring in people in the region for at least 28 years are questions that require further investigation.

Because no weekly case count data are available from outside the capitals of Angola and the DR Congo, we were restricted to modelling the potential for location-specific yellow fever virus introduction and circulation, rather than predicting cumulative numbers of expected cases and the quantitative effect of intervention strategies and climatic variables such as rainfall and precipitation. We were also further restricted to using suspected and confirmed cases, because the proportion of cases that have been laboratory tested remained small.^29^ Although our model performs well in predicting the expansion of yellow fever virus in the region, estimates might be improved by the addition of country specific real-time mobility estimates from mobile phone data or other sources, such as travel surveys. Such data could inform which populations contribute most to the invasion process and might help the targeting of intervention strategies.

Beyond local spread, there is a risk of international spread of the virus via air travel, as seen with the yellow fever virus cases that have been detected in travellers returning from Angola to China.^28^ In the present study, we refrained from extrapolating our results to regions other than the two that are currently at the core of the affected countries because evidence of overland cross-border local transmissions has, so far, only been reported between Angola and the DR Congo; frequent exchange along the major road from Uige, Angola, to Kinshasa, DR Congo, has been recorded (figure 4). Additionally, the Angolan district of Cabinda is located entirely within the DR Congo, which results in substantial population flows through the western coastal corridor of the DR Congo. Spread to other neighbouring countries such as Namibia, Zambia, or the Central African Republic might be possible but has not been recorded as part of the current outbreak.

The identification of locations expected to be invaded by the virus has broad implications for disease control, specifically for the prioritisation of where and when treatment and prevention measures would be best implemented to prevent subsequent rapid geographic spread of a pathogen.^30^ The importance of these techniques based on historical data has been shown for other diseases. However, our modelling techniques in this study allow the analysis of near real-time data to inform the control of an ongoing outbreak, constituting a methodological advance that is applicable to other diseases.

For the **WorldPop project** see http://www.worldpop.org.uk/
