# Correction to *Lancet Infect Dis* 2017; 17: 330–38

**DOI:** 10.1016/S1473-3099(19)30079-9

**Published:** 2019-04

*Kraemer MUG, Faria NR, Reiner RC Jr, et al. Spread of yellow fever virus outbreak in Angola and the Democratic Republic of the Congo 2015–16: a modelling study.* Lancet Infect Dis *2017; **17**: 330–38*—The appendix of this Article has been updated. This correction has been made to the online version as of Feb 15, 2019.

