# Supplementary material for: Spread of yellow fever virus outbreak in Angola and the Democratic Republic of the Congo 2015–16: a modelling study
Source: Lancet Infect Dis. 2017 Mar;17(3):330–8. doi: 10.1016/S1473-3099(16)30513-8 (PMC5332542; doi:10.1016/S1473-3099(16)30513-8)
Supplement: Supplementary appendix [file mmc1.pdf]

THE LANCET Infectious Diseases

Supplementary webappendix

This webappendix formed part of the original submission and has been peer reviewed. We post it as supplied by the authors.

Supplement to: Kraemer MUG, Faria NR, Reiner Jr RC, et al. Spread of yellow fever virus outbreak in Angola and the Democratic Republic of the Congo 2015–16: a modelling study. *Lancet Infect Dis* 2016; published online Dec 22. http://dx.doi.org/10.1016/S1473-3099(16)30513-8.

Supplementary information

Spread of Yellow Fever Virus outbreak in Angola and the Democratic Republic Congo 2015-2016: a modelling study

Kraemer, Moritz U.G.[$1] DPhil, Faria, Nuno R.[1] PhD, Reiner Jr., Robert C.[2] PhD, Golding, Nick[3,4] DPhil, Nikolay, Birgit[5,6,7] PhD, Stasse, Stephanie[8] MD, Johansson, Michael A.[9,10] PhD, Salje, Henrik[5,6,7,11], Faye, Ousmane[12] PhD, Wint, G.R.William[13] PhD, Niedrig, Matthias[14] PhD, Shearer, Freya M.[3] B.Sc., Hill, Sarah C.[1] B.A., Thompson, Robert N.[1] PhD, Bisanzio, Donal[3] PhD, Taveira, Nuno[15,16] Prof. PhD, Nax, Heinrich H.[17] DPhil, Pradelski, Bary S.R.[17] DPhil, Nsoesie, Elaine O.[2] PhD, Murphy, Nicholas R.[18] MPhil, Bogoch, Isaac I.[19] MD, Khan, Kamran[19,20] MD, Brownstein, John S.[21] Prof. PhD, Tatem, Andrew J.[22,23] PhD, de Oliveira, Tulio[24] PhD, Smith, David L.[1,2,25] Prof. PhD, Sall, Amadou A.[12] PhD, Pybus, Oliver G.[1] Prof. DPhil, Hay, Simon I.[2,3] Prof. D.Sc., Cauchemez, Simon[5,6,7] PhD

1. Department of Zoology, University of Oxford, Oxford OX1 3PS, UK
2. Institute for Health Metrics and Evaluation, University of Washington, Seattle, USA
3. Oxford Big Data Institute, Li Ka Shing Centre for Health Information and Discovery, Oxford OX3 7BN, UK
4. School of BioSciences, University of Melbourne, Parkville, VIC 3010, Australia
5. Mathematical Modelling of Infectious Diseases, Institut Pasteur, Paris, France
6. Centre National de la Recherche Scientifique, URA 3012, Paris 75015, France
7. Center of Bioinformatics, Biostatistics and Integrative Biology, Institut Pasteur, Paris 75015, France
8. Health Programme Manager, European Commission, International Cooperation and Development, Delegation en RDC, Kinshasa, RDC
9. Centers for Disease Control and Prevention, San Juan, Puerto Rico
10. Center for Communicable Disease Dynamics, Harvard T.H. Chan School of Public Health, Boston, USA
11. Department of Epidemiology, Johns Hopkins Bloomberg School of Public Health, Baltimore, MD 21205, USA
12. Arbovirus and Viral Hemorrhagic Fever Unit, Institut Pasteur da Dakar, Senegal

13. Environmental Research Group Oxford, Department of Zoology, South Parks Road, Oxford, OX1 3PS, UK
14. Robert Koch Institut, 13353 Berlin, Germany
15. Research Institute for Medicines, Facildade de Farmacia, Universidade de Lisboa, Lisbon, Portugal
16. Centro de Investigacao Interdisciplinar Egas Moniz, Instituto Superior de Ciencias da Saude Egas Moniz, Caparica, Portugal
17. Computational Social Science, ETH Zurich, 8092 Zurich, Switzerland
18. University of California San Francisco, School of Medicine, San Francisco, USA
19. Division of General Internal Medicine and Infectious Diseases Toronto General Hospital, Toronto, Canada
20. Li Ka Shing Knowledge Institute, St. Michael's Hospital, Toronto, Canada
21. Harvard University Medical School, Boston, USA
22. WorldPop, Department of Geography and Environment, University of Southampton, Southampton, UK
23. Flowminder Foundation, Stockholm, Sweden
24. School of Laboratory Medicine and Medical Sciences, Nelson R. Mandela School of Medicine, College of Health Sciences, University of KwaZulu-Natal, Durban, South Africa
25. Sanaria Institute for Global Health and Tropical Medicine, Rockville, USA

[$]**Corresponding author**:

Moritz U. G. Kraemer, The Tinbergen Building, S Parks Road, OX1 3PS, Oxford, United Kingdom; moritz.kraemer@zoo.ox.ac.uk; +447780336170

Calculation of the generation time distribution of yellow fever

Although epidemiological parameters of yellow fever are still poorly characterised, we can try to use field and experimental data to reconstruct the generation time distribution of yellow fever.

Human incubation period (HI)

The incubation period is the time between infection and the time of symptom onset. For the human incubation period we used a truncated exponential distribution with a mean of 4 days and a maximum time of one week.[1]

Human to mosquito transmission (HM)

We assume that the duration of infectivity of human cases is exponentially distributed with a mean of 3 days for up to a maximum of 10 days.[2]

Mosquito infectiousness (MI)

The period of mosquito infectiousness depends on the lifespan of the mosquito and the extrinsic incubation period (the time between infection in the mosquito from blood feeding of an infectious human to it becoming infectious itself and able to transmit to a new host). The average lifespan of *Aedes aegypti* is 7 days with a maximum of 30 days.[3] The extrinsic incubation period for yellow fever has been estimated at 6·9 days.[2]

Generation time distribution

We derived the empirical distribution of the generation time by simulating values for HI, HM and MI. A human case contributed to the transmission process on each day they were infectious (so the number of mosquitoes infected by a case was proportional to the duration of infectivity of the case). The same was true for mosquitoes.

Figure S1 shows the empirical generation time distribution we obtained. The generation time is estimated to have a mean of 15·0 days and a standard deviation of 5·6 days.

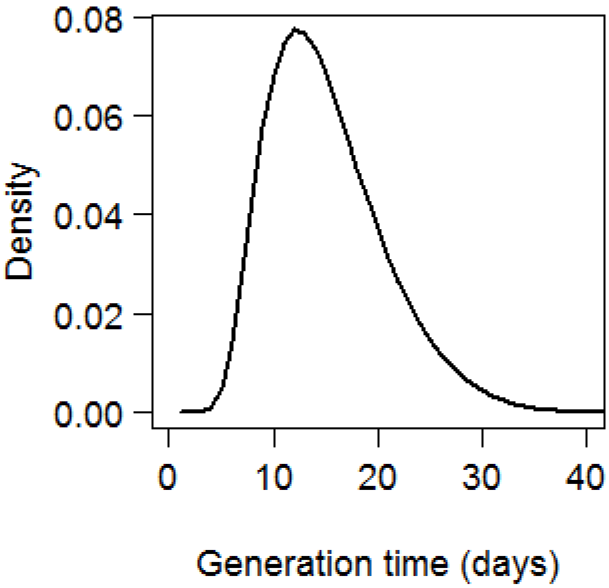

Figure S1: Empirical distribution of the generation time for Yellow Fever.

Estimation of the exponential growth rate, the doubling time and the reproduction number

Exponential growth rate

We fit a simple exponential growth rate model to the early stage of the epidemic:

$$I_w = I_0 exp(r_W.w)$$

where I_w is the number of cases on week w.

The time period for which exponential growth occurs is determined by plotting the log of the weekly number of cases (Figure S1) and selecting the time period when this variable grows linearly. A simple linear model is then fitted on this time period to estimate r_W:

$$ln(I_w) = ln(I_0) + r_W.w$$

The daily exponential growth rate r is a simple function of the weekly exponential growth rate r_W:

$$r = r_W/7$$

Between week 1 and week 5 in 2016, we estimate that the weekly exponential growth rate r_W is 0·80 (95% CI: 0·71, 0·90) and the daily exponential growth rate r is 0·11 (95% CI: 0·10, 0·13).

Doubling time

The doubling time D can be derived from the exponential growth rate r with the following formula:

$$D = ln(2)/r$$

Reproduction number

Denote $g(.)$ the density of the generation time (i.e. time lag from the infection of a case to the infection of the persons they infect). The following formula can be used to derive the reproduction

number R from the exponential growth rate r if the generation time distribution $g(.)$ is assumed to be known.[4]

$$R = \frac{1}{\int_0^\infty exp(-r.t)g(t)dt}$$

Human movement metrics:

Two generalized movement models used in this analysis were the gravity model $T_{i,j} = k\frac{N_i^\alpha N_j^\beta}{d_{i,j}^\gamma}$, and the radiation model $T_{i,j} = T_i\frac{N_i N_j}{(N_i+s_{i,j})(N_i+N_j+s_{i,j})}$, where total commuting is $T_{i,j}$ from district i, to j; N_i^α is the population in the origin and N_j^β in the destination district; $d_{i,j}^\gamma$ the distance between them, and $s_{i,j}$ the population in the radius between i and j.

Geographic spread model

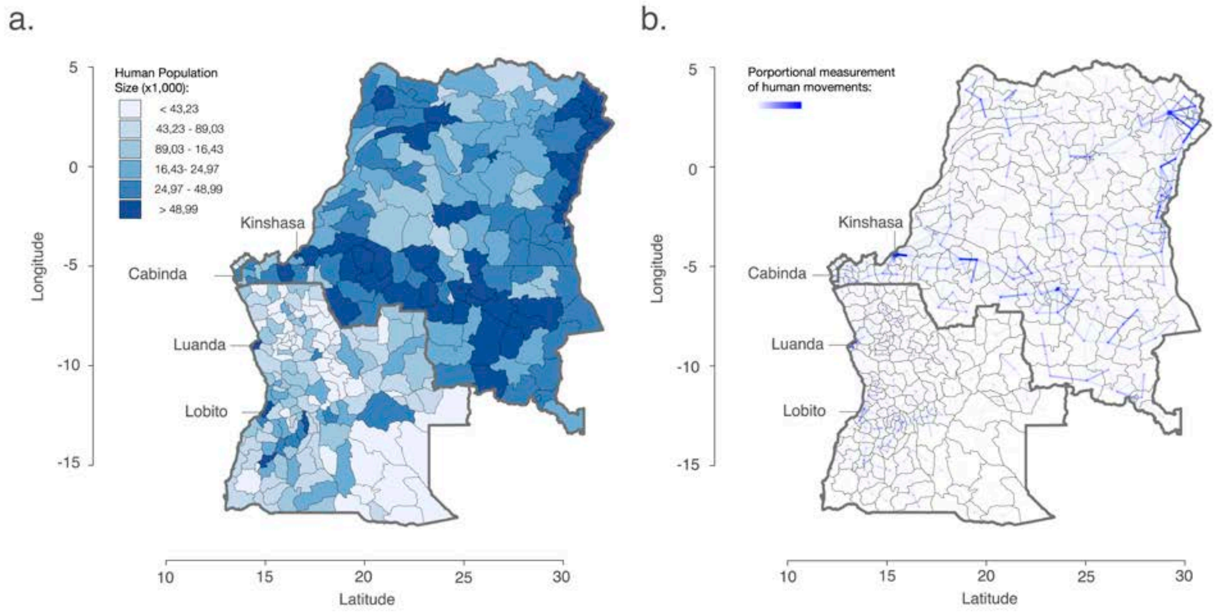

Figure S2: Population distribution in the study area (a) and relative human connectivity (b) between each district (Angola) and commune (DRC). The width of the arrows indicate the strengths of the connection.

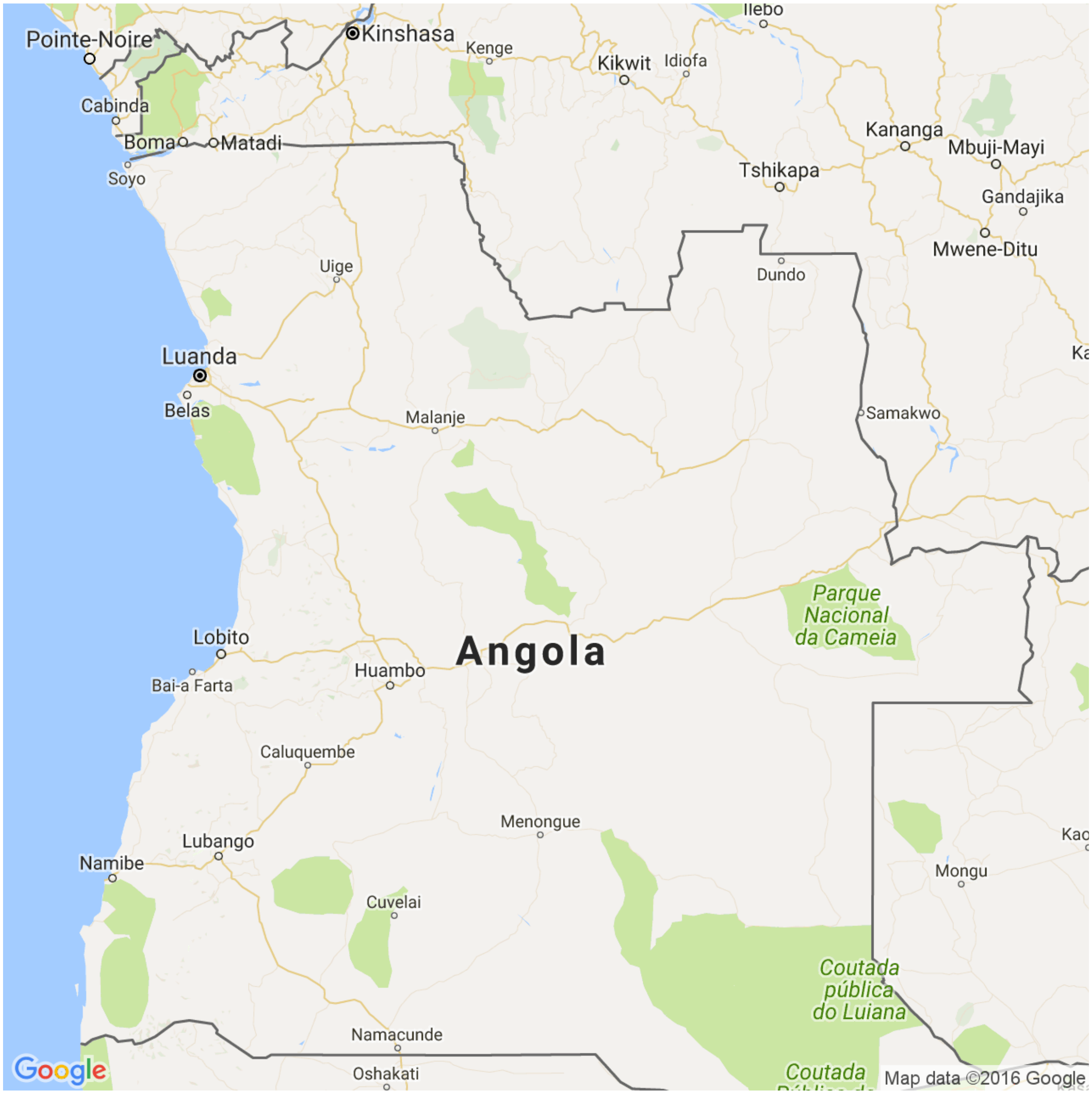

Figure S3: Road network in Angola and southern Democratic Republic Congo (www.maps.google.com).

Table S1: List of variables used in geographic spread model.

	Name	Reference
1	Great circle distance	rdist.earth function in the 'fields' package in R
2	One away adjacency	GADM shapefile (http://www.gadm.org)
3	Two away adjacency	GADM shapefile (http://www.gadm.org)
4	Three away adjacency	GADM shapefile (http://www.gadm.org)
5	Gravity model	Movement package in R, based on Zipf et al. 1946[5]
6	Radiation model	Movement package in R, Simini et al. 2012[6]
7	Uniform selection model	Simini et al. 2013[7]
8	Binary variable pre/post expansion phase	Before and after week 14
9	*Aedes aegypti* suitability	Kraemer et al. 2015[8]
10	*Aedes aegypti* suitability weighted by mobility	Kraemer et al. 2015[8], Simini et al. 2012[6]
11	Travel time distance	Uchida and Nelson 2008[9]

Supplementary Results

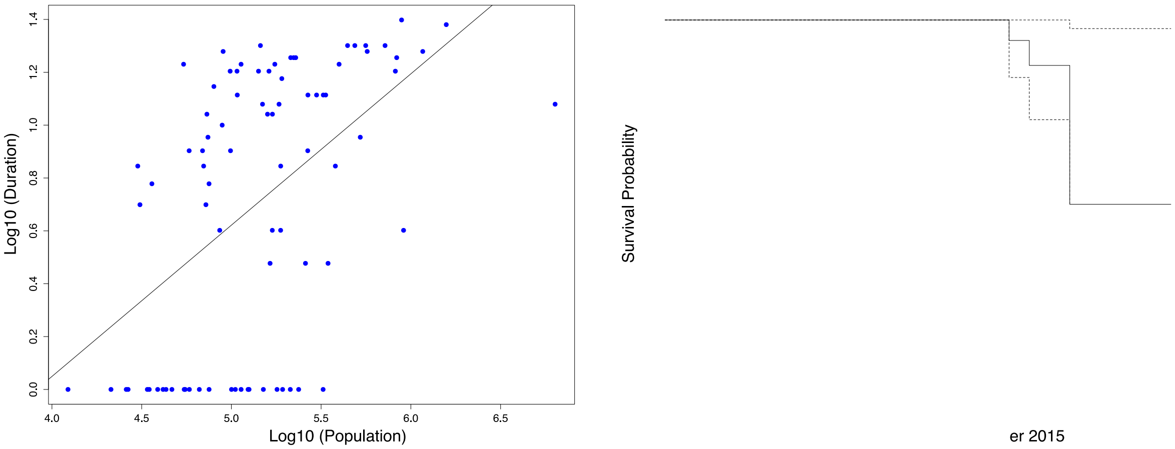

Figure S4: Results showing the relationship between population density and duration of transmission (a). Panel b) shows the results from the Cox model.

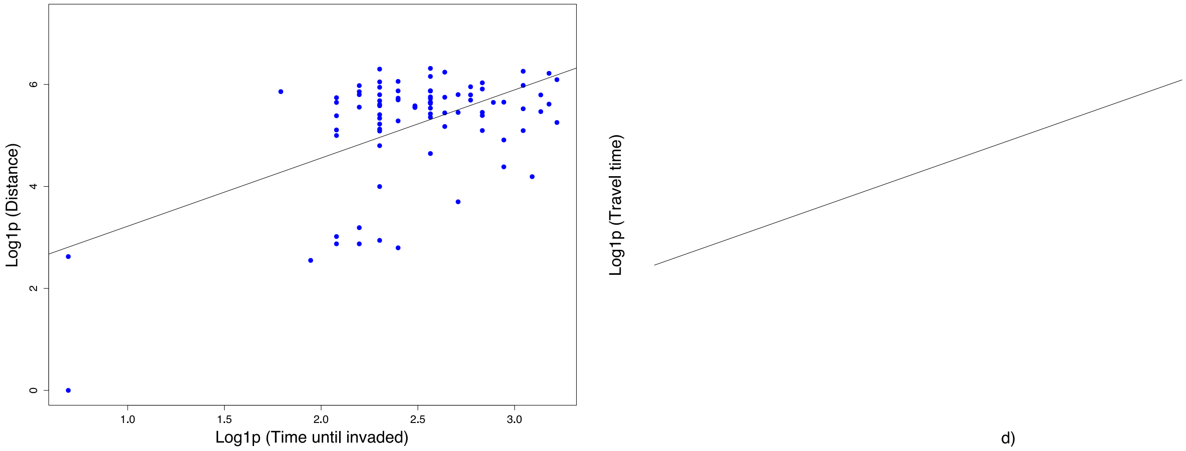

Figure S5: Relationship between distance (a), travel time (b) and time until a district was invaded.

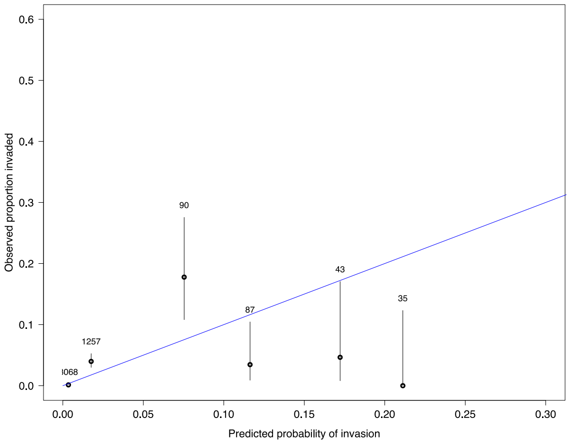

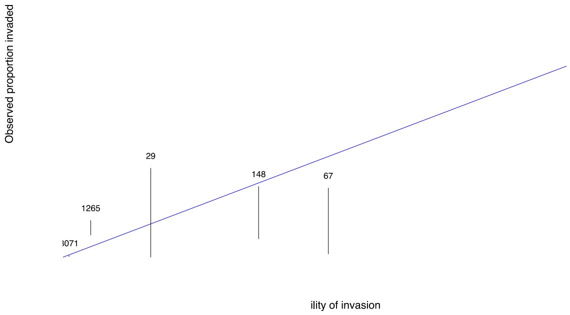

Figure S6: Comparison between observed proportion of districts invaded and their predicted probabilities using the univariate models; a) *Aedes aegypti* probability of occurrence; b) Great circle distance; c) Gravity metric; d) Neighborhood model; e) Radiation metric; f) Travel distance metric.

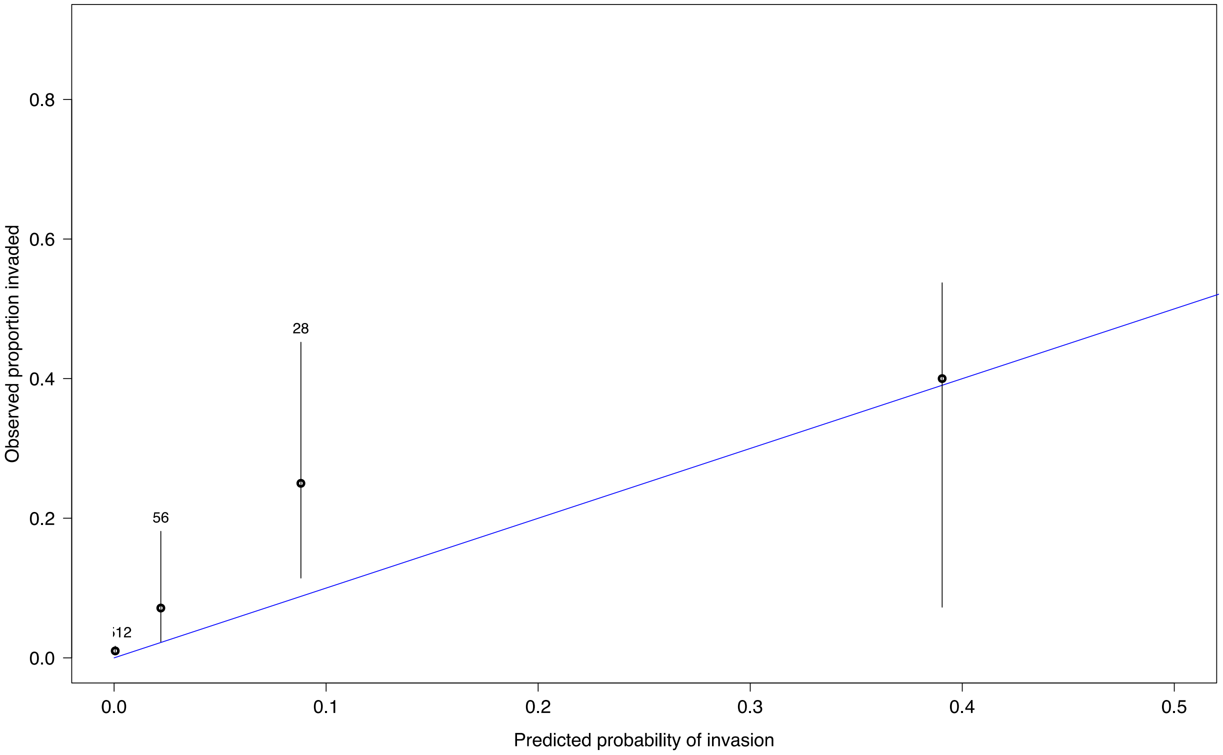

Figure S7: Model accuracy for the real-time predictions between weeks 9-12 of 2016 during the outbreak with a model using data only until week 8 of 2016. Blue line indicates perfect calibration.

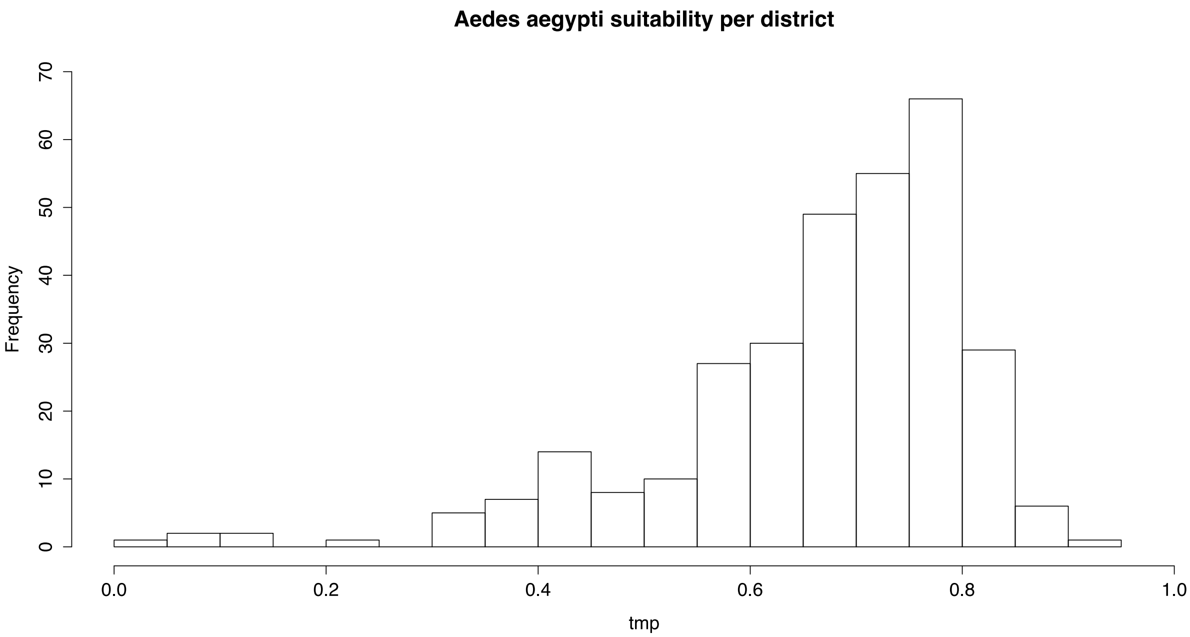

Figure S8: Histogram of district mean values of *Aedes aegypti* suitability for the study region. Estimates are taken from Kraemer et al. 2015.[8]

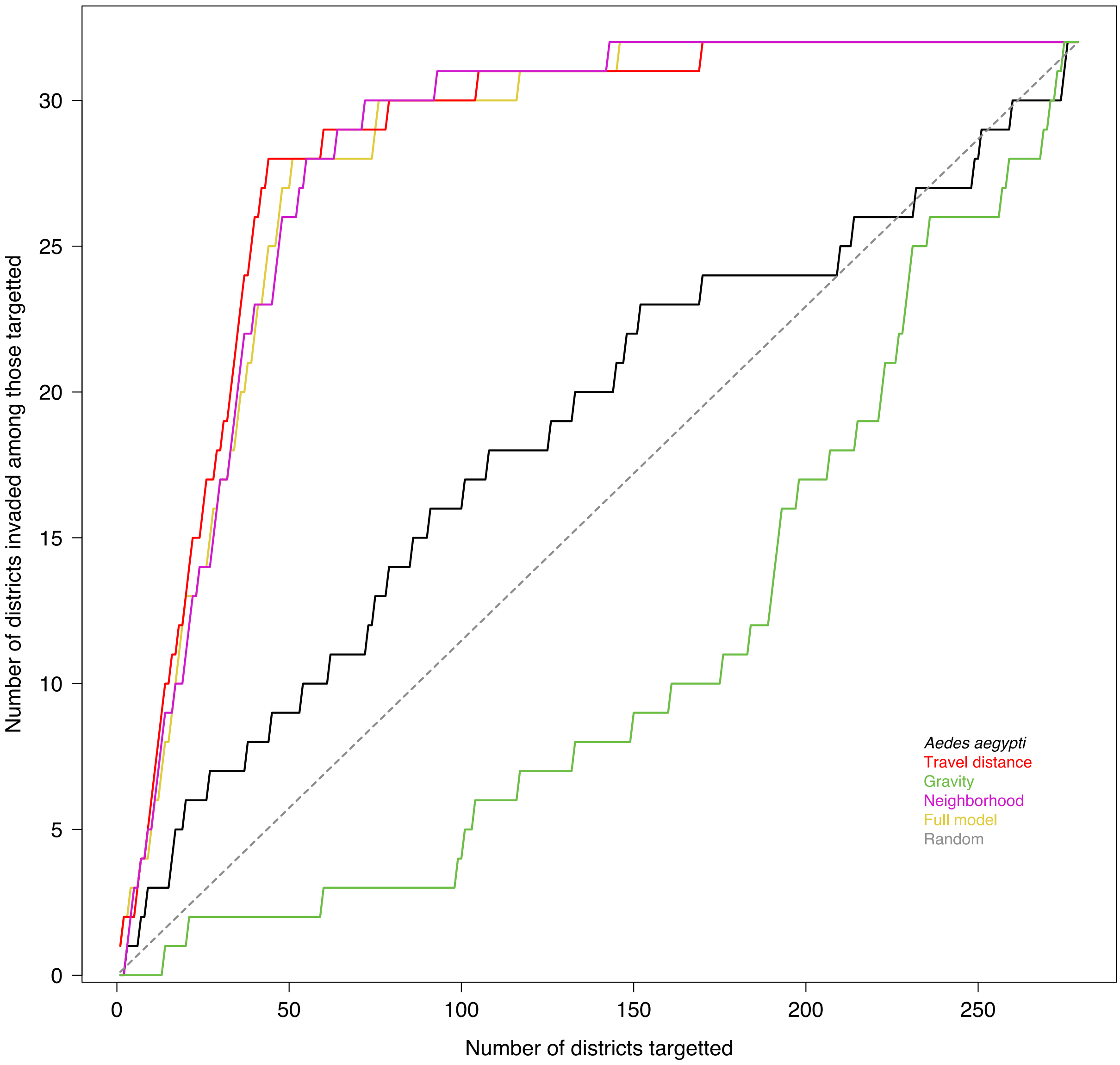

Figure S9: Model comparison of full model vs. univariate models for the expansion phase of the outbreak.

Table S2: Inclusion probability of variables in the model when using backward selection based on significance.

Name	Weeks variable was retained in the model (starting week 11 of outbreak)	Total number of weeks
Great circle distance	11, 18, 19, 21, 23, 25, 27 – 34	14
One away adjacency	14	1
Two away adjacency	15-34	20
Three away adjacency	13, 14, 16, 18	4
Gravity model	11-14, 16-18, 21-34	21
Radiation model	14, 15, 19-23, 25-34	17
Uniform selection model	11, 12, 21, 23-25, 27-34	14
Binary variable pre/post expansion phase	14-34	21
Aedes aegypti suitability	12, 13, 16-19, 21-24, 26-34	19
Aedes aegypti suitability weighted by mobility	13, 14, 16-18, 21-34	19
Travel time distance	12-17, 20	7

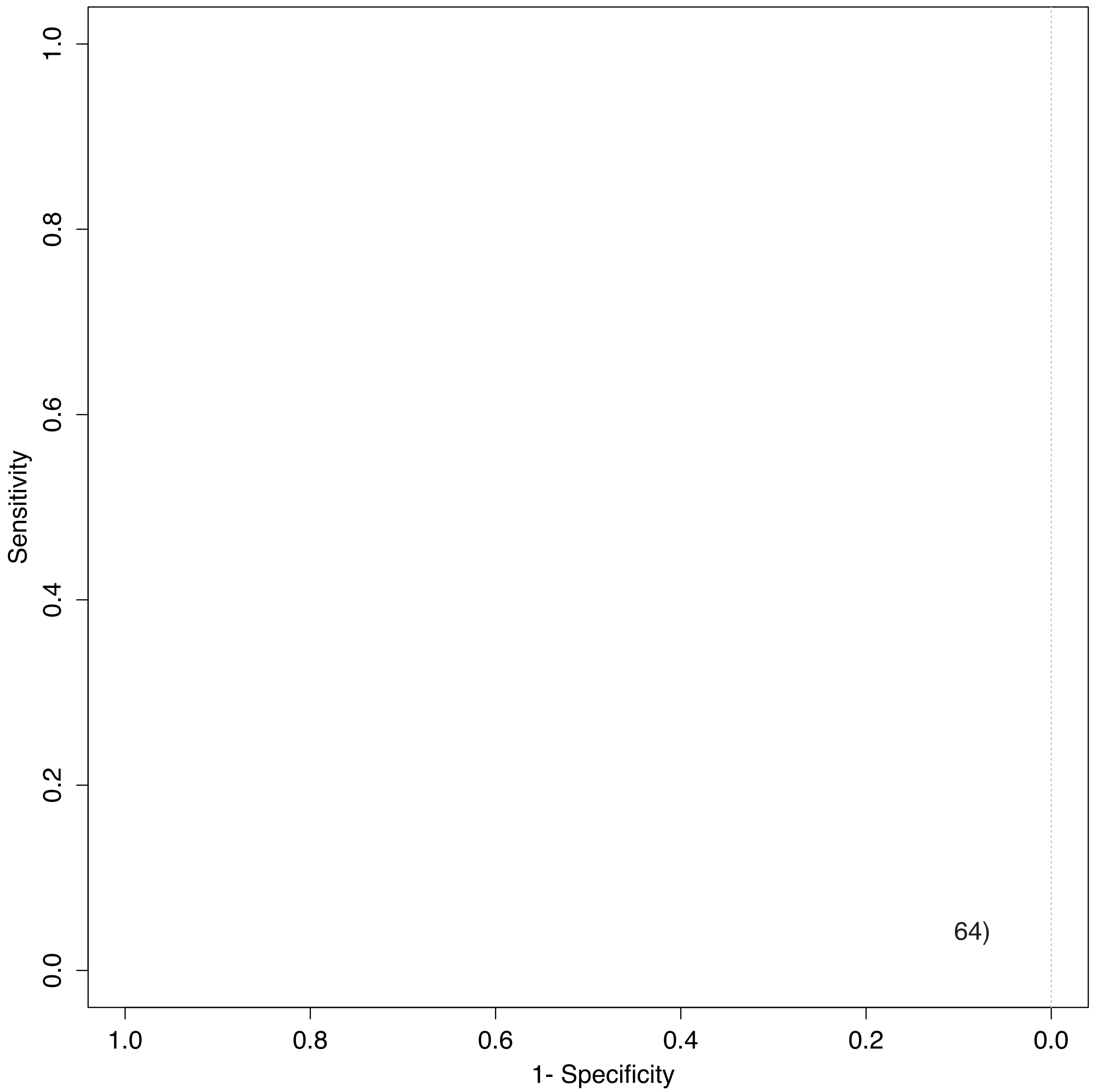

Figure S10: Predictive accuracy of the full model (green) and the full model assuming reporting delays (red) of four weeks in the early phase of the epidemic (until week 9 of 2016) and one week in subsequent weeks.

Table S3: Parameter coefficients for full model and full model assuming reporting delays of four weeks in the early phase of the epidemic (until week 9 of 2016) and one week delay in subsequent weeks.

Parametric coefficients	Covariate	Estimate	Ch.sq	Std. error	p-value
Full model	Intercept	-6.365271		0.411084	<0.001
	Three away	-0.098956		0.063441	<0.001
	Before/after intervention	-0.053673		0.007646	<0.001
	Aedes (smooth)		59.72		<0.001
	Radiation model (smooth)		33.33		<0.001
	Gravity model (smooth)		47.86		<0.001
Full model assuming reporting delays	Covariate	Estimate	Ch.sq	Std. error	p-value
	Intercept	-7.162161		0.690742	<0.001
	Three away	-0.069311		0.063506	<0.001
	Before/after intervention	0.051188		0.007419	<0.001
	Aedes (smooth)		66.73		<0.001
	Radiation model (smooth)		29.25		<0.001
	Gravity model (smooth)		40.48		<0.001

Table S4: Parameter coefficients for the full model with assumed delays of the effect of vaccination three weeks prior to implementation until five weeks after.

week of implementation	coefficient	p-value	Std error	Dev explained
-5	-0.03254	>0.05	0.05617	24.4%
-4	-0.03254	>0.05	0.01715	24.4%
-3	-0.08947	<0.001	0.01160	33.6%
-2	-0.08949	<0.001	0.01159	33.6%
-1	-0.068937	<0.001	0.008667	32%
0	-0.0537	<0.001	0.007195	30.8%
1	-0.03528	<0.001	0.005253	28.4%
2	-0.039253	<0.001	0.005377	30.1%
3	-0.040447	<0.001	0.005435	30.4%
4	-0.038561	<0.001	0.005214	30.3%
5	-0.042271	<0.001	0.005414	31.5%
6	-0.042866	<0.001	0.005569	31.7%

References

1 Johansson MA, Arana-Vizcarrondo N, Biggerstaff BJ, Staples JE. Incubation periods of yellow fever virus. *Am J Trop Med Hyg* 2010; **83**: 183–8.

2 Johansson M a, Arana-Vizcarrondo N, Biggerstaff BJ, Gallagher N, Marano N, Staples JE. Assessing the risk of international spread of yellow fever virus: a mathematical analysis of an urban outbreak in Asuncion, 2008. *Am J Trop Med Hyg* 2012; **86**: 349–58.

3 Bellan SE. The importance of age dependent mortality and the extrinsic incubation period in models of mosquito-borne disease transmission and control. *PLoS One* 2010; **5**: e10165.

4 Wallinga J, Lipsitch M. How generation intervals shape the relationship between growth rates and reproductive numbers. *Proc R Soc B* 2007; **274**: 599–604.

5 Zipf GK. The P1 P2 / D hypothesis: on the intercity movement of persons. *Am Sociol Rev* 1946; **11**: 677–86.

6 Simini F, González MC, Maritan A, Barabási A-L. A universal model for mobility and migration patterns. *Nature* 2012; **484**: 96–100.

7 Simini F, Maritan A, Néda Z. Human mobility in a continuum approach. *PLoS One* 2013; **8**: e60069.

8 Kraemer MUG, Sinka M, Duda K, *et al.* The global distribution of the arbovirus vectors Aedes aegypti and Ae. albopictus. *Elife* 2015; **4**: e08347.

9 Uchida H, Nelson A. Agglomeration index: towards a new measure of urban concentration. Washington D.C., 2008.
